# Rheumatology: Necessary adjustments to the realities of the new era in Greece

**DOI:** 10.31138/mjr.28.2.94

**Published:** 2017-06-27

**Authors:** Panagiotis I. Trontzas

**Affiliations:** Greek Rheumatology Society, Athens, Greece

**Keywords:** Rheumatology, precision medicine, patient rights, education, health services

## Abstract

Rheumatic Diseases (RDs) are a serious, though often not sufficiently recognized, problem; strongly impacting not only patients’ socio-economic activity but their quality of life in general. Yet, despite the tremendous progress made over the past few years, several questions, in regard to better management of people with rheumatic conditions, remain unanswered. Furthermore, many institutional problems and fixations in doctors’ and patients’ culture pose additional obstacles to the best treatment of these diseases. In Greece specifically, there are numerous and serious structural problems in the system of providing health services to people with rheumatic diseases; as well as in the education, professional training and development of Rheumatologists, which have been aggravated even more by the prolonged economic crisis. The scientific rheumatological community, and particularly its institutional representatives, need to implement a long-term plan for the correct and documented application of modern methods for the diagnosis, treatment and support of people with rheumatic diseases. They also need to lead the effort for the creation of a culture of cooperation between the parties concerned; namely the various professional groups of rheumatologists, other health professionals, patient associations and the state.

## BACKGROUND

Rheumatology is a medical specialty that deals with the study, diagnosis and treatment of the systemic rheumatic and other musculoskeletal diseases. There are more than 200 rheumatic diseases (RDs), which, apart from the joints, may also affect internal organs; leading to significant morbidity, functional disability and mortality. In addition, they are accompanied by numerous and considerable comorbidities.

It is established that 1/4 of the population in developed countries would suffer from a RD at some point in their lives. Within the European Union (EU) people with RDs amount to 120,000,000, ^1^ while in Greece they are estimated to be about 3,000,000. ^2^

RDs affect overall quality of life, as they hinder physical activity more than Diabetes, Cancer and Cardiovascular diseases. They limit social interactions, cause disabilities and reduce life expectancy. ^3^ For example, serious, active Rheumatoid arthritis reduces life expectancy by 10–15 years. ^4^ In Greece, RDs are the leading cause (among all illnesses) of chronic health problems (38.7%), long-term and short-term physical disability (47.2% and 26.2% respectively) and medical visits (20.5 %), while they are ranked as the second cause for consumption of prescription and non-prescription drugs (24.0% and 17.7% respectively). ^5^

RDs are internationally included among major diseases that weigh on all the socioeconomic structure, and the EU classifies them as “significant” diseases. Due to their nature and clinical course, as well as their impact on patients’ quality of life and daily routine, these diseases are accompanied by high costs for the health care system, the patient and society. ^6–8^ According to the World Health Organization and the European League Against Rheumatism (EULAR) estimates, the total cost of RDs amounts to 1.2% – 3% of a country’s GDP in developed economies. ^9–10^ It is also estimated that for every 1 euro of the direct cost for treating a patient with a RD, an additional 1–2 euros should be accounted for indirect expenses and costs, such as the value of goods that are lost (not produced) due to the illness and the patients’ need to be cared for by third parties; usually family members. ^11^ Finally, there is the considerable social cost of RDs, affecting the development and prosperity of society, and straining its cohesion. Patients suffer severe consequences on their personal, family, professional and social life. ^12^

## I. THE CLINICIAN’S VIEW

Since the advent of the new millennium, massive new knowledge on inflammation has been acquired (mechanisms, responsible molecules, cells, homeostatic regulation of the body) resulting in the development of targeted specific therapies for inflammatory rheumatic diseases. Rheumatologists now possess an abundance of knowledge arising from basic research and its clinical application. They are able to set an early diagnosis and to readily implement the indicated treatment, ensuring the immediate alleviation of suffering and the long term prevention of the disease’s destructive course. ^13^ Hence, on one hand, resources are spared, as patients do not use the health services consecutively and needlessly (clinical examinations by doctors who do not recognize the illnesses, inappropriate laboratory tests and treatments), and on the other hand, the complications of the diseases, which add high costs to the health systems, are prevented; while, at the same time, patients’ quality of life and productivity are improved.

The rheumatologist can now use new specialized drugs, such as macromolecular biologic agents, in order to treat inflammatory arthritis, systemic autoimmune diseases, familial Mediterranean fever, resistant gout, osteoporosis, but also micro molecular targeted disease modifying drugs for treating scleroderma, systemic lupus erythematosus, rheumatoid arthritis, psoriatic arthritis and gout. These treatments, although extremely expensive, have proven cost-effective as they result in a large and rapid reduction in symptoms, inhibit the radiological development of diseases, prevent disability, and drastically improve the patients’ work productivity and general quality of life. ^14^

Nevertheless, the current therapeutic goal for rheumatic diseases, “complete remission”, remains a challenge. A small percentage of patients, both in clinical trials and in real life, achieve remission or even low disease activity. ^15^ But even for those who achieve the goal, the rate of relapses is very high after discontinuation of treatment. For patients who do not initially respond to the treatment, or experience undesirable side effects, the strategies of cycling or switching drugs are applied. Finally, for those who simply have a good level of therapeutic response, the effect only lasts for a few years, and then the changing of the drugs of the same or an alternative therapeutic pathway begins. ^16^

All of these are done in a rather random way, since there are still no reliable biomarkers ^17,18^ for the choice of the appropriate drug for the specific patient (personalized medicine), the biologic tapering strategies are still unclear, ^19^ and the patients’ compliance with the treatment is not satisfactory. ^20^ Thus, although emphasis is now placed on the timeliness of the diagnosis and the early immunotherapy, in reality, a lot of valuable time is lost for most patients before they receive the drug that is appropriate for them. Reliable and documented answers are needed to be given soon to the following questions:

- Which drug is suitable for which patient?
- What exactly is the successful therapeutic response?
- What is the precise definition of remission?
- How long does the treatment last, when and how should it be discontinued?
- What is the best practice on failure of treatment?
- How much, and in what way, should the patients’ preference for treatment be taken into account, to improve compliance and the end result?

## II. THE PRESIDENT’S VIEW

For RDs to be effectively dealt with, however, apart from addressing the existing scientific insufficiency, certain institutional adjustments must also be implemented, and changes must occur in the culture and attitudes of the stakeholders. The proper management of RDs requires a harmonious cooperation in the “patient - health professionals - state” triangle. The perceptions and objectives of each entity are quite diverse due to the distance among them and the inequality of the roles that prevailed until recently. The EU, after a EULAR proposal, has adopted since 2010 a specific strategy for the care of patients with RDs, and its principles are described in the Brussels Declaration on Rheumatic and Musculoskeletal Diseases 2010: ^1^

1. ***Recognition:*** *The European Union and its Member States should recognise the socioeconomic importance of rheumatic and musculoskeletal diseases of all ages and assign them appropriate priority.
*
2. ***Research:*** *There is an urgent need to prioritise basic and clinical research regarding the causes, predictors, management and impact of these chronic diseases.
*
3. ***Reintegration:*** *The European Union and Member States should ensure that people with disabilities related to rheumatic and musculoskeletal diseases have the right to full inclusion in society: this encompasses optimisation of environmental and life-style factors, the availability of self-management tools and respect for the right to a flexible education and work environment.
*
4. ***Quality health care:*** *People with rheumatic and musculoskeletal diseases should receive prompt access to high quality care, ideally in specialised centres, thus maximising long-term quality of life.
*
5. ***Evidence-Based Medicine:*** *Management of rheumatic and musculoskeletal diseases should be in accordance with evidence-based recommendations in every European Union Member State.
*
6. ***Rights:*** *People with rheumatic and musculoskeletal diseases are experts in living with their condition and should be involved in the design, delivery and evaluation of their services.
*

### IIa. The present situation in Greece

Before, but particularly during my presidency of the Greek Rheumatology Society and the Professional Association of Rheumatologists (ERE-EPERE, 2015–2016), I had the opportunity to personally identify the diversity and plethora of problems for both patients with RDs and rheumatologists. Regarding the management of rheumatic diseases in Greece there are:

*
- Unbalanced distribution of rheumatology health services.
*
*
- Unwise distribution of the limited resources.
*
*
- Minimal knowledge, specialization and availability of allied health professionals (specialist nurses, physiotherapists, occupational therapists, psychologists, etc.).
*
*
- Total lack of cooperation between the public and private health sectors.
*
*
- Bureaucracy and difficulties in accessing the necessary services.
*
*
- Managing disability by providing benefits and allowances.
*
*
- Total lack of social reintegration policies for RDs patients.
*
*
- Deficiency in the doctors and patients’ perception of the importance of adherence to treatment.
*
*
- Inadequate information and training of both doctors and patients on the value and implementation of cost-effectiveness in medical procedures.
*
*
- Limited consent by rheumatologists to accept the guidance and control of medical procedures on the basis of the Evidence-Based Medicine principles.
*
*
- Fragmentary, arbitrary and often non-scientific efforts by the state to control medical procedures (tests, treatments).
*
*
- High degree of dependence and regulation of the continuing medical education by the pharmaceutical industry.
*
*
- Discord in the relationships among the different professional groups of rheumatologists and also among the various associations of patients with RDs.
*

Above all, however, there is suspicion and total absence of co-operation of everybody with everybody. What dominates are stereotypical and obsolete perceptions, authoritarian ruling, union dependence and interconnections, and maximalist demands, whereas the lack of planning from all sides is quite obvious.

Despite all that, ERE-EPERE, considering that the prolonged economic and social crisis that is affecting Greece could become an opportunity for radical change, took the initiative and, in co-operation with the Patient Associations and specialized scientists, has designed a long-term **“Action Plan for the Rheumatic Diseases”** , ^21^ which includes seven thematic axes of actions on:

- The necessary reforms in the system and in health policies
- Informing and raising public awareness of rheumatic diseases
- Creating registry records of people with RDs
- Developing support and care programs for chronic RDs patients, especially those who belong to vulnerable population groups (uninsured, economically weak, people with disabilities and residents of geographical areas that are hard to reach)
- The development of volunteering to cover complementary needs
- The training of doctors, other health professionals, and of patients and their families
- The promotion of RDs research (epidemiology, aetio-pathogenesis, clinical research, production of Greek financial data for the burden to the health system)

The experience from the procedures of designing and promoting the “Action Plan” highlighted the multiple problems of cooperation. The effort to create a common perception among the different parties was tedious and slow. Competition and the maximalistic attitude of the patients’ associations were continually undermining the endeavour. The established doctor-centric culture of most rheumatologists has been acting as a deterrent. Above all, however, it is the suspicion, inaction, and lack of co-operation on the part of the state, and the institutional bodies, that threaten the whole project with peril.

Meanwhile, in recent years, ERE-EPERE has taken bold initiatives on the formulation and implementation of guidelines and on the electronic application of therapeutic and diagnostic protocols in the e-prescription system. The effort has not always been successful, particularly in the field of diagnostic protocols; due mainly to resistance, ingrained perceptions, and the ill-organized processes of the responsible institutional bodies. The same factors hindered the advancement of the program for the development of national “Registries” for people with rheumatic diseases and their treatments. An additional reason for this last failure was the lack of willingness for co-operation within the rheumatology community.

Another important issue is training and education across different rheumatology centers in the country. Currently, Greek rheumatology trainees perform the major part of their specialty training - 4 years - in the same department which inevitably represents an important limitation. In addition, nonstandard procedures for the appraisal and validation of both trainers and trainees have been established and specifically for supervisors there are no training programs. The discussion has just opened, and issues such as the application of a standard curriculum including a minimum number clinical assessments and procedures, the determination of specialty positions per department and the accreditation of educational centers able to provide high-standard training based on strict criteria are extensively analysed. Although the process of discussing and shaping different approaches and views among all stakeholders, including university departments, centers of the national health system and clinicians themselves is difficult and strenuous, the implementation of high quality standards and the harmonization of specialty training is essential not only to support the development of Rheumatology in Greece but more importantly to facilitate equal standards of care for patients with RDs in the country. ^22^

### IIb. The future

There is no other choice but to keep going forward. The demands of the new era will only be met with long-term planning, partnerships and innovative initiatives. An illustrative example of what cooperation can achieve is the very journal that hosts this article. The Mediterranean Journal of Rheumatology (MJR), with its new, international and modern format, is an accomplishment, and once it is embraced by the research centers of the region, it will have a bright future ahead.

We need to organize a new and decisive strategy, characterized by:

- Understanding the overall context and the difficulties.
- Mapping the infrastructure and identifying the needs of the health system.
- Adhering to the patients’ needs, particularly with regard to their regaining their normal life and reintegrating into the social, work and family environment.
- Paying attention to the needs of health care practitioners, particularly in terms of recognizing their role and enhancing their opportunities for continuing education and professional development.

The Greek scientific rheumatological community has the obligation to inspire and lead in the effort of changing the current state of affairs, by focusing on the following topics:

- *
Adoption, promotion and implementation of the actions included in the “Action Plan for the Rheumatic Diseases”, in cooperation with the Patient Associations, institutional agents and specialists.
*
- *
Creation of reliable registries for rheumatic diseases (patients and illnesses).
*
- *
Completion and constant updating of the therapeutic and diagnostic protocols of rheumatic diseases.
*
- *
Finalization of the e-health system by creating the necessary and also user-friendly applications.
*
- *
Implementation of the required reforms in specialty education and in continuing education in Rheumatology.
*

Concurrently, ERE-EPERE, as the official institutional representative of Rheumatology in Greece, should reform and reconstruct itself on new bases. To better fulfill its contemporary role, it could be restructured into distinct departments of activity, such as:

- Specialty training
- Conferences and continuing education
- Research
- Evidence-based medicine and protocols
- Professional issues (it already exists)
- Communication and relations
- Institutional interventions
- Promotion of the “Action Plan”
- Development of social programs for the support of RDs patients
- Constant flow of information to the general public and awareness-raising of Rheumatic Diseases

These departments would need to be staffed with paid personnel, and have administrative support. This is where the society should invest its resources, to produce useful and meaningful work, continuously assessed against international scientific criteria. The success of the project would require the different professional groups of the rheumatological community to be fully and constantly involved through their distinct roles: academics, rheumatologists of the public sector and those self-employed - because the future is common to all, and everyone is going to benefit from the “spring” of Rheumatology in this small but historic Mediterranean region. However, it should never be forgotten that the rheumatological community’s purpose of existence and its main objective is the treatment and care of people with rheumatic diseases, thus it is not entitled to ever break free from this primary value framework.

## CONCLUSION

RDs have high burden, which is now recognized and treated to a significant extent with targeted therapies. We still need further research to achieve prevention, personalized medicine, steady regression, complete remission and, ultimately, healing and cure. We also need, through an interdisciplinary approach, fostered social awareness and institutional protection, to produce better, higher quality and more complete health care services for people with rheumatic diseases.

The rheumatological community must embrace the new perception for the economic evaluation of medical procedures, accept the gradual change from the medical-oriented to a society-oriented model, and cooperate effectively with state authorities, to organize health systems based on the actual needs and social priorities.

Finally, the scientific rheumatological societies should lead the effort of adapting to the demands of the new age, undertake the responsibility for the training of rheumatologists and other health professionals, promote research, and provide guidance and support to people with RDs, their families, their associations and society at large, with the singular goal of eliminating morbidity, disability, social exclusion, and ultimately attaining a sustainable quality of life for people with Rheumatic Diseases.
